# Artificial intelligence workflow quantifying muscle features on Hematoxylin–Eosin stained sections reveals dystrophic phenotype amelioration upon treatment

**DOI:** 10.1038/s41598-022-24139-z

**Published:** 2022-11-19

**Authors:** Marie Reinbigler, Jérémie Cosette, Zoheir Guesmia, Simon Jimenez, Catalin Fetita, Elisabeth Brunet, Daniel Stockholm

**Affiliations:** 1 Télécom SudParis, Institut Polytechnique de Paris, 91120 Palaiseau, France; 2 Généthon, 91000 Evry, France; 3 Centre de Recherche en Myologie, UMR-S 974, Institut de Myologie, 75000 Paris, France; 4 École Pratique des Hautes Études, PSL University, 75000 Paris, France

**Keywords:** Computational science, Diagnostic markers, Image processing, Machine learning, Gene therapy

## Abstract

Cell segmentation is a key step for a wide variety of biological investigations, especially in the context of muscle science. Currently, automated methods still struggle to perform skeletal muscle fiber quantification on Hematoxylin-Eosin (HE) stained histopathological whole slide images due to low contrast. On the other hand, the Deep Learning algorithm Cellpose offers new perspectives considering its increasing adoption for segmentation of a wide range of cells. Combining two open-source tools, Cellpose and QuPath, we developed MyoSOTHES, an automated Myofibers Segmentation wOrkflow Tuned for HE Staining. MyoSOTHES enables solving segmentation inconsistencies encountered by default Cellpose model in presence of large range size cells and provides information related to muscle Feret’s diameter distribution and Centrally Nucleated Fibers, thus depicting muscle health and treatment effects. MyoSOTHES achieves high quality segmentation compared to baseline workflow with a detection F1-score increasing from 0.801 to 0.919 and a Root Mean Square Error (RMSE) on diameter improved by 31%. MyoSOTHES was validated on an animal study featuring gene transfer in $\gamma$-Sarcoglycanopathy, for which dose-response effect is visible and conclusions drawn are consistent with those previously published. MyoSOTHES thus paves the way for wide quantification of HE stained muscle sections and retrospective analysis of HE labeled slices used in laboratories for decades.

## Introduction

Muscular dystrophies are a group of genetic diseases in which the impairment or the absence of muscle proteins can lead to severe phenotype causing muscle cell death, often associated with a loss of muscle function. Muscular dystrophies also share, at various degrees, histological patterns at cellular scale. In particular, those pathologies imply a process of degeneration and regeneration of muscle fibers, that can be quantified by measuring fibers size and the Centrally Nucleated Fibers (CNF) ratio in muscle sections^1–4^. Such parameters are crucial not only for characterizing muscle dystrophy progression but also for establishing and evaluating therapeutic approaches. Those measurements have been widely carried out on immunofluorescent labeled sections because of their high contrasts, making those images suitable for segmentation by former image processing tools^5–12^.

Artificial intelligence greatly opens up perspectives. Beyond enhancing the exploitation of immunofluorescent labeled muscle sections, it now enables to process less contrasted standard Hematoxylin-Eosin (HE) stained sections, that is an omnipresent, easy-to-use and inexpensive labeling protocol, on future studies and on the huge unexploited archived studies banks because of manpower lack. In this paper, we present MyoSOTHES, standing for Myofibers Segmentation wOrkflow Tuned for HE Staining. MyoSOTHES is a generalist workflow tuned to quantify dystrophic markers, i.e. fiber size and CNF ratio on HE stained histology sections. For this purpose, after data preconditioning, muscle sections are segmented thanks to the state-of-the-art deep learning algorithm Cellpose^13^, prior to be quantified using the QuPath bioimage analysis tool^14^.

MyoSOTHES muscle-tuned workflow precision was evaluated on a manually determined ground truth for which the F1-score is enhanced from 0.801 obtained with the generalist baseline workflow to 0.919 and the Root Mean Square Error (RMSE) on diameter is 31% lower than the best performing *default* model configuration. Moreover, for the sake of wide usage, the computational consumption of MyoSOTHES has been taken into consideration in terms of memory footprint and execution time. MyoSOTHES does not require more than 2 GB of memory, i.e. 2 GB of RAM (Random Access Memory) in Central Processing Unit (CPU) mode only and 2 GB of Graphics Processing Unit (GPU) memory with the combination of CPU and GPU modes. On desktop machines, it remains executable in average measured computation time of about 14 min 44 s and in 4 min 55 s on machine equipped with GPU to analyze muscle section images being on average 120MB each.

We also validated MyoSOTHES on a cohort of whole slide images (WSI) of HE stained psoas sections (muscle of pelvic belt) from mice suffering from $\gamma$-Sarcoglycanopathy, and treated with different doses of gene therapy vectors. We showed that our analysis reveals the dose-effect and is consistent with the previous study conclusions^15^.

## Results

To conduct HE stained muscle histological image analysis, we built a baseline workflow based on two main tools: Cellpose^13^, which is a generalist deep-learning algorithm for cellular segmentation, and QuPath, a visualization and analysis tool for histological images. As depicted in Supplementary Fig. S1, this baseline workflow consists in three stages. To get started, WSI can be easily visualized and selected in QuPath for analysis. They are then exported via QuPath in TIFF format, compatible with the next workflow stage. Once in TIFF format, WSI are segmented using Cellpose. Output of this stage are PNG images of masks corresponding to each cell detected in the input image. As a last step, the set of predicted masks are imported into QuPath as regions of interest, which are superimposed on corresponding section for further analysis and are processed to perform quantitative analysis. In muscle distrophy context, we target to measure phenotype parameters as the fiber Feret’s diameter distribution and CNF ratio estimation using nuclei detection plugin of QuPath.

To evaluate the reliability of the segmentation stage and finally of the overall workflow results, the segmentation detection quality is quantified by measuring the F1-score, as described in Section “Methods” and depicted in Supplementary Fig. S2, by superimposing ground truth hand-made segmented images and Cellpose masks predicted on those same images. Similarly, the segmentation quality has been verified by comparing predicted mask diameter and corresponding hand-made mask diameter measuring RMSE, as referred in Section “Methods”.

**Figure 1:**
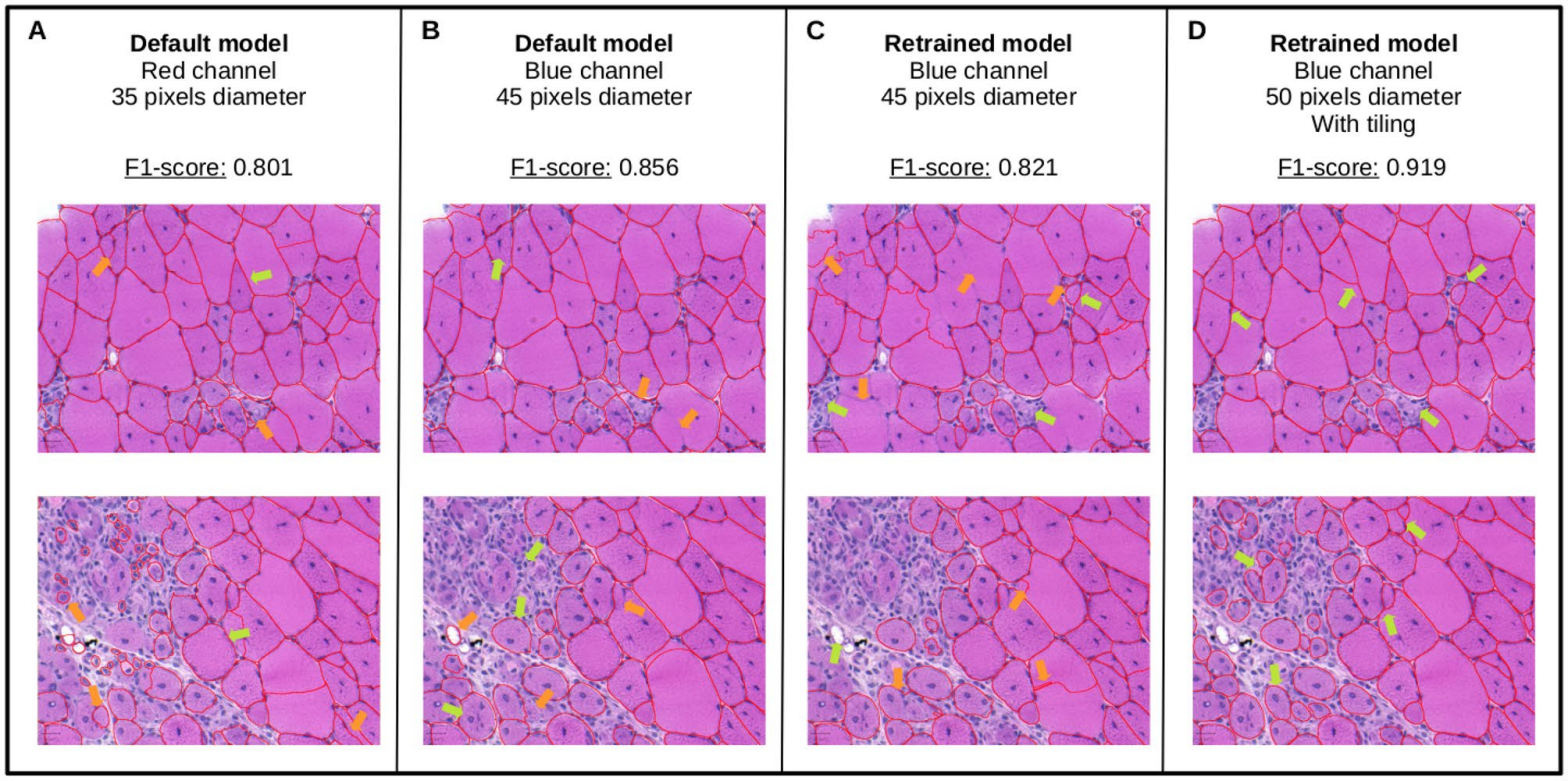
Representative results of segmentation using the different workflow advances. (**A**) The *default* model with the initial parameters gives a 0.801 F1-score. (**B**) The *default* model with tuned parameters has an increased F1-score of 0.856. (**C**) The *retrained* model has promising results in the areas where the muscle is highly damaged (green arrows), but has an unsatisfactory 0.821 F1-score, mainly due to misdetection of large fibers (orange arrows). (**D**) The *retrained* model coupled with tiling of images reaches a F1-score of 0.919, by keeping satisfactory detection in damaged area, and restoring detection of large fibers.

With a F1-score of 0.801 (see Fig. 1A), this baseline workflow leaves room for optimizations to reach the muscle-tuned MyoSOTHES. In this respect, we can act at several levels: before computing Cellpose segmentation by introducing a pre-processing step; at Cellpose level, by acting on its parameters and configuration; and after Cellpose segmentation, by selecting relevant produced masks. The incremental optimization strategies we explored to tune this baseline workflow in order to reach the muscle-tuned MyoSOTHES are presented hereafter.

### Cellpose parameters tuning

Cellpose^13^ is an algorithm composed of two main building blocks. The first block is a deep learning algorithm, whose architecture is derived from the widespread U-net architecture^16^. Its model has been trained as following: each ground truth cell has been intermediately represented by the topology map of a heating diffusion source starting from the center of the mask. This topological map includes horizontal and vertical gradients. Those gradients, together with the probability to belong to a cell, are the model’s predicted outputs. The second building block consists in combining horizontal and vertical gradients. The direction of the gradient is followed towards the central point common to other pixel gradients whose pixel probability to belong to a cell was predicted higher than 0.5. All pixels whose pixel gradient points to the same center constitute one cell mask. The output recovered is a PNG image where pixels belonging to the same cell mask have the same value.

In this work, Cellpose^13^ is used as a black box that enables our workflow to segment the WSI. The only parameters we can control are the image color channel, initially selected to be the red one, and the estimated cell diameter, initially equal to 35 pixels. Therefore, we explored different sets of parameters by varying both the image channel and the cell diameter. To discriminate between best detecting model, we measured the diameter RMSE with respect to the ground truth to keep the model with both best quality and detection ability. As summarized in Table 1, the best configuration turns out to be the blue channel together with a cell diameter of 45 pixels which led to a F1-score improvement from 0.801 to 0.856 for a RMSE estimated to 4.27 $\upmu$m.

### Cellpose specialization

As we can see in Fig. 1A,B, the segmentation obtained is accurate in healthy areas where muscle fibers have well delimited and regular shapes, whereas segmentation quality drops in inflammatory areas (green arrows), in areas with a high heterogeneity in fiber size and with elongated cells (orange arrows). To address this problem, we took advantage of Cellpose 2.0^17^ new feature that enables to retrain Cellpose ground model using fine-tuning techniques to specialize it to our specific dystrophic muscle dataset. The retraining was performed on nine patches extracted from our dataset with as largest diversity as possible on blue channel, as it revealed to be the most effective according to Table 1. During the retraining process (refer to Section “Methods”), there were always a trade-off to make between large cell segmentation and small fibers segmentation. Once a trade-off seemed reached for training patches, the retraining is stopped. The model was then tested on a whole slide image not used for retraining at highest resolution with input cell diameter of 45 pixels. Note that, interestingly, despite good segmentation performance of fine-tuned model achieved at patch-level during training in comparison to baseline model with previously selected set of parameters, the F1-score reached for whole slide image is 0.821, which is lower than the *default* model with blue channel and 45 pixels cell diameter setting. Indeed, Fig. 1A–C shows that the *retrained* model misses a lot of well delimited fibers, including areas where it performs well at patch-level. To alleviate this problem, we propose to explore the overwriting of Cellpose tiling strategy by adding an image tiling pre-processing step and a WSI segmentation reconstruction post-processing step. The efficiency of this optimization strategy will be assessed in comparison to the use of this same retrained model using only Cellpose internal tiling strategy.

**Table 1:** Optimization of cellpose *default* model input parameters.

	Set of parameters (diameter, channel)			
	(50, red)	(45, blue)	(60, green)	(50, gray)
RMSE ($\upmu$m)	4.45	4.27	4.35	4.66

The RMSE of diameters with respect to the ground truth for *default* model is measured for models with a set of parameters leading to the best detection ability for each channel. The selected configuration is the combination of diameter 45 and blue channel.

**Table 2:** Optimization of *retrained* model input parameters using tiling strategy.

Diameter ($\upmu$m)	Channel			
	Red	Blue	Green	Gray
**F1-score**				
30	0.827	0.832	0.828	0.844
35	0.881	0.882	0.879	0.895
40	0.891	0.908	0.895	0.908
45	0.899	0.915	0.910	0.908
50	0.893	**0.919**	0.914	0.900
55	0.900	0.912	0.910	0.909
60	0.896	0.910	0.903	0.905

The cell diameters are varied together with channel color. The F1-score is measured for each configuration to identify the best set of parameters, highlighted in bold. Finally, a diameter of 50 pixels is selected with a channel set to blue.

### Segmentation pre-processing: image tiling

The segmentation by image tiling is a three-step process, which starts before applying Cellpose. The first phase consists in dividing the WSI into tiles and to analyze only those belonging to tissue bounding boxes in order to limit computation time. For now, areas containing relevant tissue are detected using a QuPath thresholder. Once this area is delimited, it is divided into patches of 712 $\times$ 712 pixels with an overlap with neighbour patches of 200 pixels to ensure no fiber mask is cut by both overlapping patch limits (see Supplementary Fig. S3). Then, each tile is individually segmented by Cellpose, using the *retrained* model with same parameters. Once the individual tile masks obtained, the whole WSI masks are reconstructed, as described in Section “Methods” and illustrated on Supplementary Fig. S4.

The tiling phase enabled to enhance the F1-score from 0.821 to 0.915. To achieve the best F1-score possible using tiling strategy, the choice of the most adapted cell diameter for *retrained* model was evaluated using same method as for the *default* model and revealed to be 50 pixels coupled with blue channel (see Table 2). This choice of cell diameter enabled to reach a F1-score of 0.919. The improvement introduced by tiling with most adapted cell diameter is clearly visible on Fig. 1D. Areas with regular fiber size and shapes are as well segmented while in inflammatory and heterogeneous areas, small fibers are more often detected. In addition to a detection improvement, it turns out retraining enabled to also improve segmentation quality as RMSE decreased from 4.27 $\upmu$m for selected *default* model to 2.94 $\upmu$m, meaning a 31% improvement.

**Figure 2:**
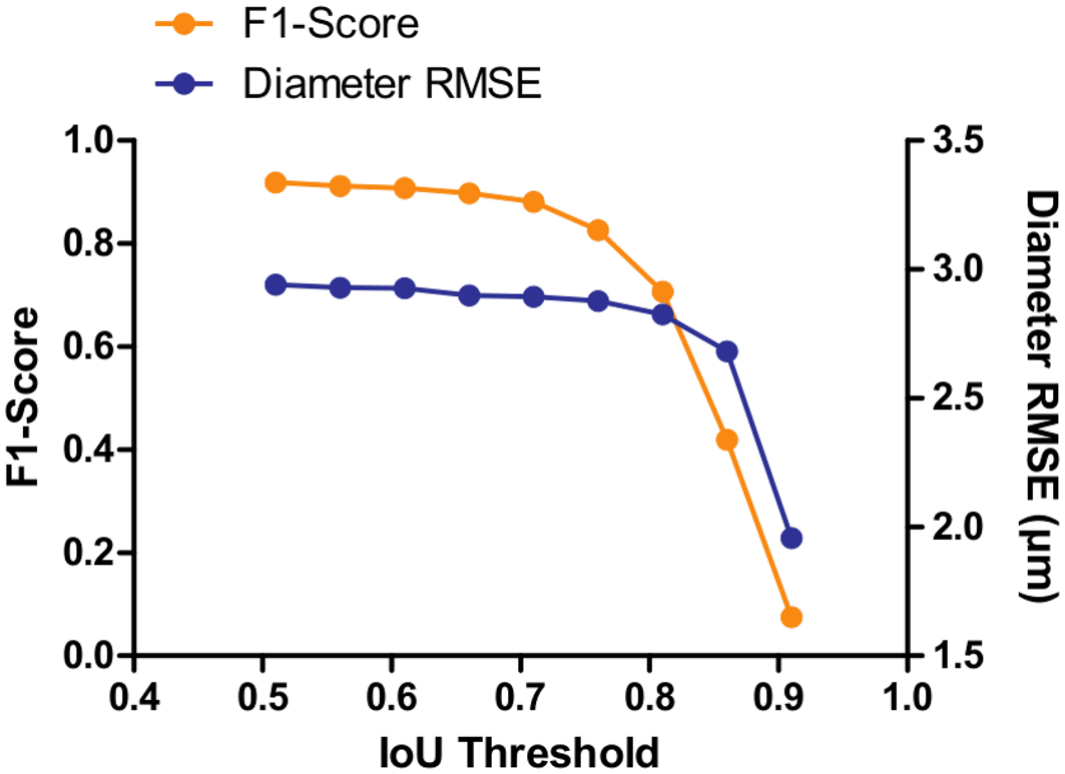
Evaluation of IoU threshold impact on F1-score, meaning model detection ability, and diameter RMSE in $\upmu$m, indicative of model segmentation quality. Increasing the IoU from 0.51 to 0.71 introduces a negligible impact on detection capability of the algorithm. Similarly, an increased IoU from 0.51 to 0.81 introduces a small impact on segmentation quality. From 0.76 threshold, the F1-score decreases quickly until reaching almost 0 for an IoU higher than 0.91. From 0.86 threshold, the RMSE reduces significantly of about 30%.

**Figure 3:**
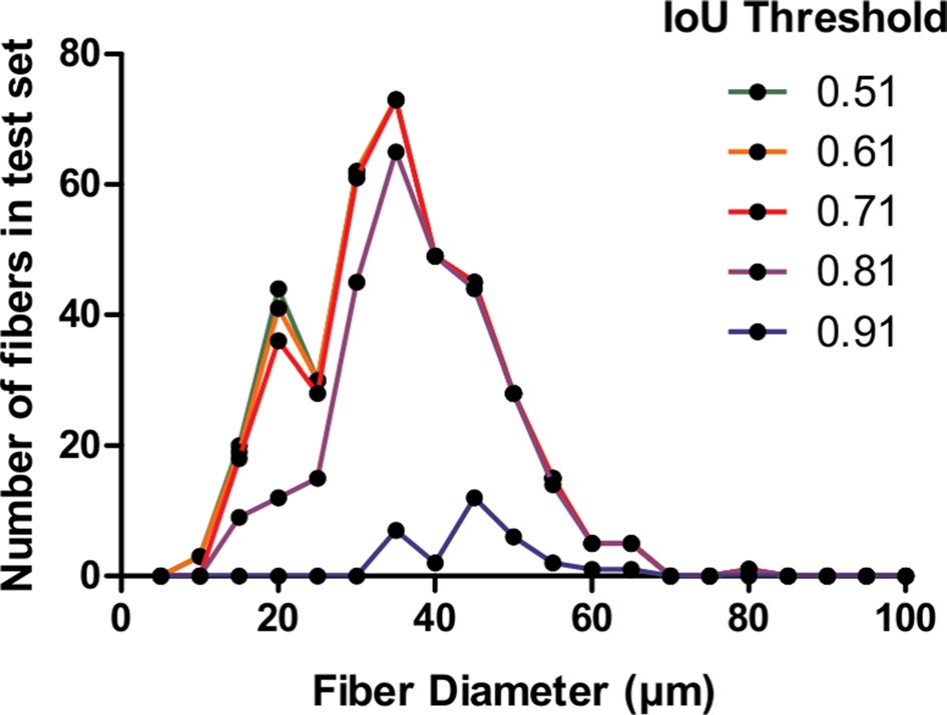
Impact of IoU threshold on fiber diameters class retained. From IoU higher than 0.71, the distribution is biased: the largest and smallest fibers tend to be rejected.

All F1-score and RMSE measurements have been performed considering an Intersection over Union (IoU) threshold higher than 0.5 as commonly used in famous segmentation challenges such as the Coco Challenge^18^ or the Voc Challenge^19^. To ensure our good results do not come solely from a low IoU threshold, the influence of IoU threshold on F1-score and diameter RMSE was further investigated. Figure 2 indicates that starting an IoU threshold higher than 0.71, the F1-score drops until reaching a value close to 0. In parallel, the RMSE is getting improved from about one third. By looking at the histogram, such degradation in detection ability introduces a bias in the cell population considered for analysis as diversity of cell diameter available decreases with the threshold increasing. Indeed, on test set, the number of fiber diameter classes of a range of 5 $\upmu$m is 12 with an IoU of 0.71, whereas there are only 7 left with an IoU threshold of 0.91, with the removal of smallest and largest diameters, as underlined by Fig. 3. For IoU threshold from 0.51 to 0.71, the RMSE remains quite stable, with a degradation of 1.4%, representing 0.04 $\upmu$m of difference, which is negligible in comparison to the microscope error estimated to 1 $\upmu$m. On the other hand, the F1-score gets degraded by 4.3%. Consequently, selecting a IoU threshold of 0.51 enables to keep a high detection capacity, thus avoiding biasing the considered cell population, while only slightly degrading the RMSE. Following these observations, an IoU threshold of 0.51 is the one used for every other measurements and analysis performed in this paper.

### Segmentation post-processing: artifacts filtering

The Cellpose output can be improved by applying a filter removing segmentation artifacts, that are the masks which do not correspond to cells. Indeed, as Cellpose consists in computing pixel gradients and reconstructing cell masks based on gradient direction convergence, it cannot differentiate between cell borders and artifact borders. Two categories of artifacts can be encountered. Some artifacts take their origin in histology processing. They can be tears in the section coming from the cutting phase, black spots corresponding to dust or bubbles. Examples are referred in Supplementary Fig. S5. The second category of artifacts corresponds to any biological element present in the muscle section that does not correspond to muscle fiber. For example, artifacts could be blood vessels, lymphatic vessels or connective tissue. In this case, there is no common shape characteristics between all those artifacts, however they differentiate from cells by their colors. Thus, a pixel classifier based on a Random Forest algorithm^20^, has been built using parameters listed in Section “Methods”. This ensures that the biological parameters to be measured based on segmentation will be only influenced by the fibers characteristics.

### MyoSOTHES: resulting workflow

**Figure 4:**
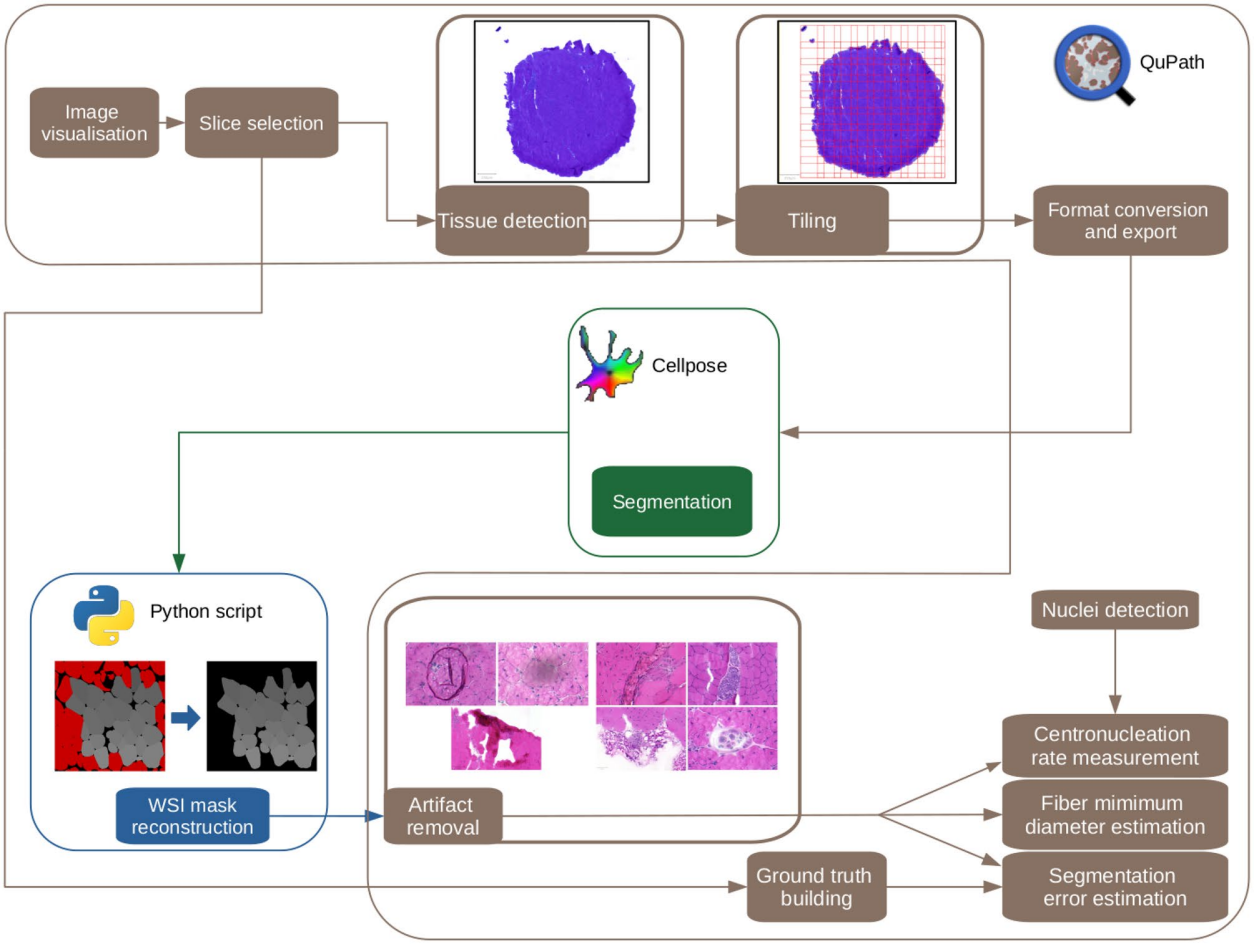
MyoSOTHES overview. After applying a pixel classifier to detect tissue edge, input WSI is tiled, and each tile is processed with Cellpose (retrained model—blue channel—50 pixels diameter). A Python custom script is then applied to the Cellpose output masks to reconstruct the total mask image of the tissue section. A final pixel classifier is then used to remove the masks corresponding to artifacts areas (fold, bubbles, dust, etc.). The masks are used to measure the fibers Feret’s diameter, and estimate the error based on ground truth manual segmentation. Segmentation masks are also combined with the position of nuclei to identify centrally nucleated fibers and measure the CNF ratio.

Based on segmentation quality estimation measure detailed in Section “Methods”, the configuration selected is the *retrained* model with an input cell diameter of 50 pixels, preceded by a tiling step, followed by a mask filter of type ‘pixel classifier’ as this configuration is the one reaching highest F1-score, low RMSE and also performing the best according to visual verification. Indeed, the *retrained* model has the advantage to not introduce segmentation noise, to detect less nucleus masks in inflammatory areas, and to segment less artifacts. The tuned workflow MyoSOTHES is summed up in Fig. 4.

### Computation performance assessment

We measured Cellpose computation time for two models: the *default* model which corresponds to the initial Cellpose 1.0 model with as input selected parameters the red channel and a cell diameter of 35 pixels—but without any retraining and tiling; and the *retrained* model which corresponds to the Cellpose 2.0 model retrained on our data with tiling and selected parameters as previously described.

Computation performance assessment has been conducted on three different platforms. The first computing platform, further referred to as M1-CPU, is composed of an Intel Core i7-10710U 1.10GHz processor, with 6 cores and 32 GB of RAM. The second computing platform, further referred to as M2-CPU, is equipped with an Intel Core i5-8400 2.80GHz processor with 6 cores and 32 GB of RAM. The third platform, further referred to as M3-GPU, is composed of 2 AMD EPYC 7502 2.5GHz processors with 32 cores and 512 GB de RAM and a Nvidia Quadro RTX 5000 GPU with 16 GB of memory.

For the ‘default’ version, we measured the mean Cellpose execution time over all WSIs of the dataset, each computed five times on each platform. For the ‘retrained’ tiled version, we summed the computation time of each tile to get the computation time of a WSI. As for the ‘default’ version, we averaged the computation time of all dataset WSIs of five iterations done on each platform. For sake of precision, each WSI is in average 120 MB.

**Figure 5:**
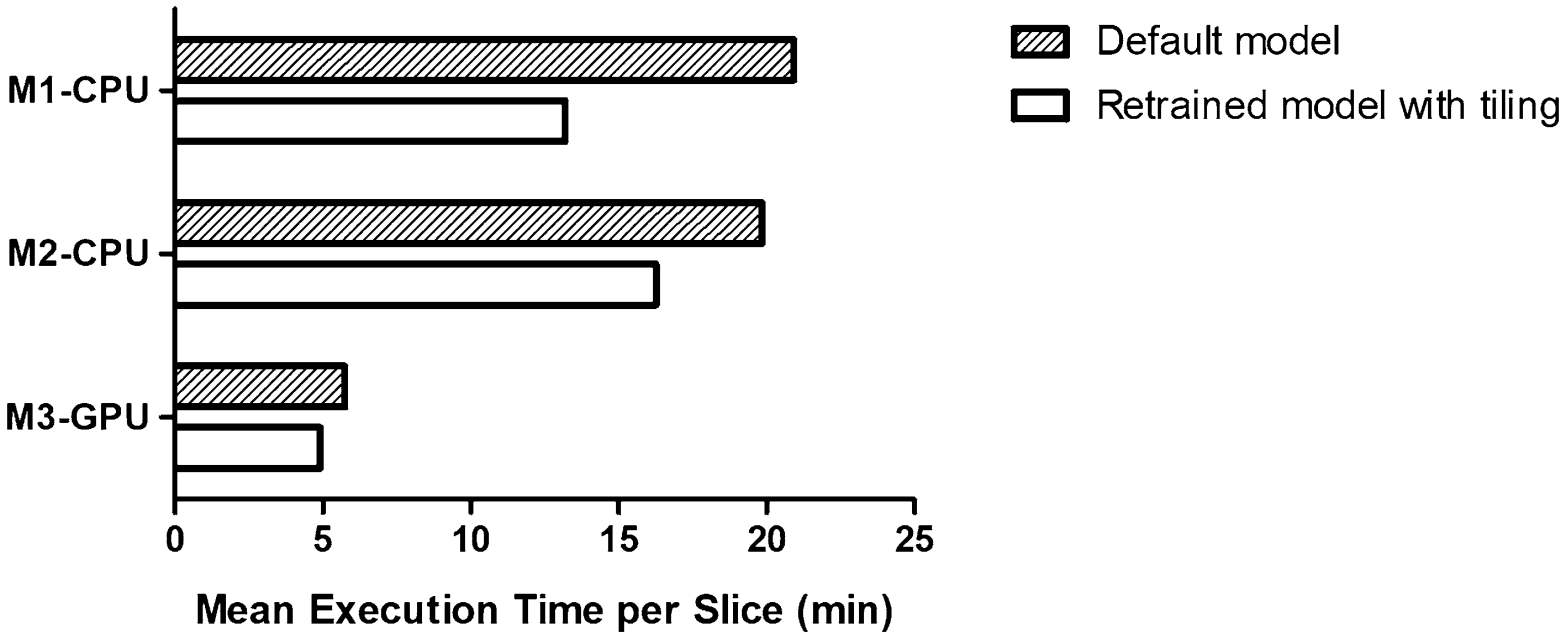
Segmentation computation time using different hardware configurations. M1-CPU designates a machine composed of an Intel Core i7-10710U 1.10GHz processor, with 6 cores and 32GB of RAM. M2-CPU is equipped with an Intel Core i5-8400 2.80GHz processor with 6 cores and 32GB of RAM. M3-GPU is composed of 2 AMD EPYC 7502 2.5GHz processors with 32 cores and 512 GB de RAM and a Nvidia Quadro RTX 5000 GPU with 16 GB of memory.

The summary of results obtained is available in Fig. 5. Running only on CPU, whether on M1-CPU or M2-CPU, the average computation time is about 20 min per WSI for the *default* model and about 15 min with the *retrained* one. On M3-GPU machine, the average mean computation time per WSI is 5 min and 45 s for the *default* model, meaning four times faster than running on CPU; and of 4 min and 55 s for *retrained* model using tiling strategy, meaning about one third of CPU-only mode computation time. This highlights the temporal benefit introduced by using a GPU. It is also remarkable that the mean computation time per WSI remains stable for the five runs.

During our experiments, we also noted the high computation footprint of the *default* model workflow. Indeed, it requires at least 32 GB of RAM and 16 GB of GPU memory to execute Cellpose in CPU-mode and GPU-CPU-mode respectively for the non tiled version. On the other hand, the memory impact is drastically lowered using tiling strategy as only 2 GB of memory are now required, both for CPU and CPU-GPU modes. In addition, tiling enabled reducing computation time, especially for CPU-only executions where it has been reduced of one-third.

### MyoSOTHES validation on a dystrophic muscle treatment study

MyoSOTHES was applied to 50 WSIs of psoas sections of 50 mice captured in the biological use-case of the study on $\gamma$-Sarcoglycanopathy published in 2018^15^. The WSIs are classified in 5 categories: 10 WSIs corresponds to Wild Type (WT) subjects, 10 to Knockout (KO) subjects, 10 received the highest treatment dose, 10 the intermediate dose, and 10 the lowest dose. Based on resulting segmentation, we expect to detect the dose-response effect on muscle phenotype using CNF ratio and Feret’s diameter as phenotype indicators.

**Figure 6:**
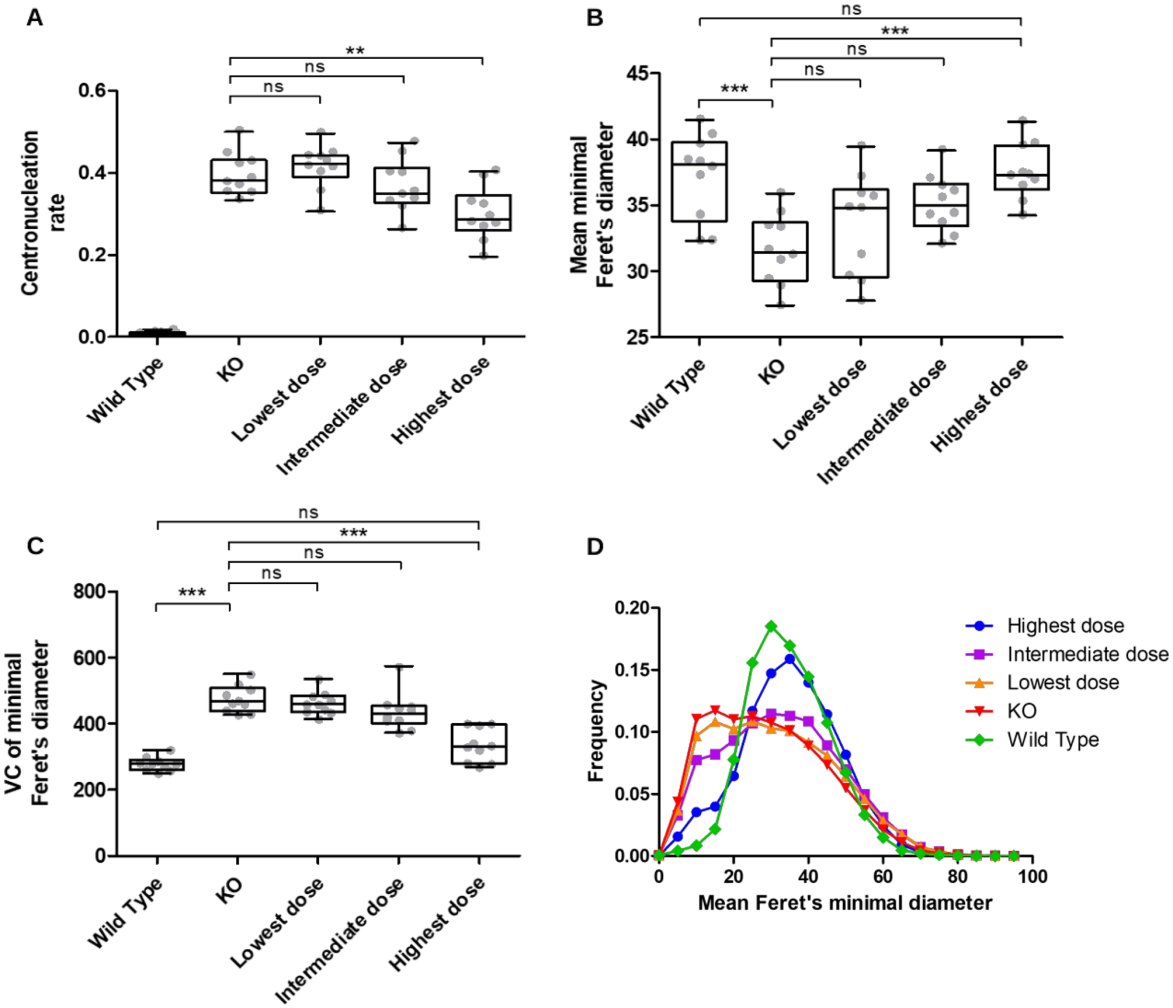
Quantification of fibers diameters, variation coefficients and CNF ratio for 50 WSI of psoas sections. Box plot (including overlaid individual dots, each corresponding to one mouse) of CNF ratio (**A**) p-value = $6\times 10^{-4}$, mean minimal Feret’s diameters (**B**) p-value = $1 \times 10^{-4}$, and variance coefficient (**C**) p-value < $1\times 10^{-4}$, for each treatment condition. Statistic test one-way ANOVA—with Bartlett’s test for variance equality and Bonferroni’s multiple comparison post test. As expected, wild type mice have very few centrally nucleated fibers. However, the highest dose condition has a significantly lower CNF ratio than the KO condition. The wild type and the Highest dose conditions are significantly different from the KO condition for both mean minimal Feret’s diameter, and their variance coefficient. Overall, for those three parameters, increasing dose of vectors tends towards phenotype recovery and exhibits a dose response effect. (**D**) Distribution of mean Feret’s diameter per condition. KO mice have a higher number of small fibers, resulting in a characteristic distortion of the distribution shape. The increasing vector dose tends to reshape the distribution towards the wild type distribution shape, as the highest dose distribution is close to the wild type distribution.

The analysis of Fig. 6A highlights the phenotype difference between WT subjects and KO subjects: the CNF ratio of the KO subjects is significantly higher, as a healthy muscle fiber has its nuclei located at its periphery. A high CNF ratio is thus a good indicator of anomaly, characteristic of myopathies^21^. The effect of the dose injected is also visible, as the higher the dose, the lower the CNF ratio. The dose-response effect is also visible on Feret’s diameters diagram on Fig. 6B). The mean Feret’s diameter of the WT and KO subjects are significantly different with a higher diameter for healthy subjects. The mean Feret’s diameter of highest dose is close to the WT one and significantly higher than the KO one. Lowest and intermediate dose groups are not statistically different from KO group, but intermediate dose seems to show small improvement, while being missing for lowest dose.

The variance coefficient (VC), drawn on Fig. 6C, confirms response-dose effect. The difference between WT and KO phenotype is depicted by a significantly higher coefficient of variance for KO subjects. This is explained by a high number of fibers being regenerated in a dystrophic muscle and thus having smaller diameters, which induces an increased heterogeneity in fiber diameters. The dose-response effect is shed into light by a coefficient of variance of highest dose subjects significantly different from the KO one and not significantly different from the WT one. This is again the only dose that statistically shows a phenotype improvement. In parallel, the intermediate and lowest doses are not significantly different from the KO one, even if the medium dose seems to tend towards a lower variance coefficient and thus confirms the tendency towards slightly better condition.

The histogram of Feret’s diameters (see Fig. 6D) reinforces these observations. The KO distribution is flatter with a shift of the peak to the left compared to the WT. This is due to the increase of the small fiber population due to regeneration. This shift is reduced within the treated subjects. The flatter the distribution and the larger the standard deviation, the lower the dose injected. The KO, lowest and intermediate doses tend to have distributions with similar shapes, whereas the highest dose tends to have similar distribution as the WT. The highest dose seems to be the only dose which sufficiently improves muscle health to have a phenotype tending towards WT phenotype, while the other two dose phenotypes seem to remain close to KO one. These results show that the minimal Feret’s diameter of fibers is a better reflection of the muscle recovery compared to the CNF ratio. The highest dose gives a CNF still highly distinguishable from the WT while the minimal Feret’s diameter mean or VC is not different statistically. Such discrepancy can be explained by the fact that regenerated fibers tend to keep their nuclei at the center of the fibers for months even though the fiber is completely mature, making the CNF a long-lasting phenotype.

The conclusions drawn in this section are valid despite the diameter RMSE of about 2.94 $\upmu$m. Indeed, the diameter RMSE of KO and WT subjects are similar, 2.95 and 2.93 $\upmu$m respectively. In addition, their RMSE distributions according to diameter class of 5 $\upmu$m from 0 to 100 $\upmu$m are not significantly different in accordance to Kolmogorov–Smirnov test whose p-value is of 0.20, which is higher than 0.05.

## Conclusion and discussion

In this work, we describe MyoSOTHES, a tuned workflow designed for quantifying skeletal muscle fiber diameters and CNF on whole slide images of HE stained sections to assess muscle dystrophy. This workflow integrates the generalist Deep Learning algorithm Cellpose, designed for cellular segmentation, which we adapted for myofibers recognition. It is coupled with open-source analysis software QuPath for biological parameters quantification. MyoSOTHES demonstrates its improved performance compared to baseline workflow when evaluating the segmentation quality against manually determined ground truth with an F1-score increasing from 0.801 to 0.919 and RMSE improved of 31% in comparison to best performing *default* model configuration. In order to be widely used, a particular focus was laid on computing consumption. MyoSOTHES has a low memory footprint with no more than 2GB of RAM required and performs on desktop machines with an execution time of less than a quarter-hour for muscle section images of 120MB in average.

Moreover MyoSOTHES is a fully automated workflow, an ideal feature to manage projects with large amount of whole slide images to analyze. However, it enables the interactive check of the segmentation quality and the spatial distribution of the parameters directly within QuPath at each stage. Finally, we validated our approach on a cohort of whole slide images of HE stained sections from mice suffering from $\gamma$-Sarcoglycanopathy, and treated with different doses of gene therapy vectors. We showed that our analysis reveals the dose-effect and is consistent with the previous study conclusions. Thus, while HE stained section are usually used for a qualitative assessment of muscle health, MyoSOTHES allows going further by offering quantitative information on the dystrophic phenotype for this staining which has for a long time represented real challenge for image analysis.

In terms of short-term enhancements, first, we encountered some difficulties with the segmentation of special myofibers cross-sections that had particularly long shapes. It could be interesting to retrain the Cellpose model to make it more efficient for these types of forms. Then, some improvements could also be obtained with some image pre-processing steps. Indeed, although MyoSOTHES is really efficient to exclude regions of targeted artifacts thanks to an integrated pixel classifier of QuPath, it may be necessary for some studies to upgrade this part of workflow to include new types of artifacts. In addition, a color normalization step could improve even more Cellpose segmentation capabilities.

To go further, from a computational point of view, our tiling method enables the use of reasonable memory GPU opening the perspective of frugality and the even wider usage. From a data perspective, pre-clinical studies and clinical studies led in pharmaceutical industry often tend towards fully automated processes to reduce user dependence and increase reproducibility. MyoSOTHES is suitable with this kind of approaches as the workflow is automated and the HE staining is suitable with automated staining devices. Our MyoSOTHES workflow thus paves the way for wide quantification of HE stained muscle section, and retrospective analyses as HE staining has been used in laboratories for decades, specially when considering numerous rare diseases for which samples can be capitalized and used for further analysis beyond their lifetime.

## Methods

### Animal and histology procedures

The human g-Sarcoglycan gene under the control of the desmin promoter (AAV8-desm-hSGCG) was encapsulated in recombinant adeno-associated virus vector (rAAV) using the protocol described in the previous study^15^. The g-Sarcoglycan KO mouse model ($Sgcg^{-/-}$) used in this study has been previously described^15^. All mice were handled according to the European guidelines for the human care and use of experimental animals, and all procedures on animals were approved by the Généthon’s ethics committee under the numbers CE10-122, CE10-123, CE10-124, CE10-127, and CE12-039. A volume of 100 $\upmu$L/20 g containing the AAV vector was injected into the tail vein. Mice are divided into 5 groups: a wild type group, a knockout group and three groups with different levels of treatment at $4.5 \times 10^{12}$ vg/kg, 1.5 $\times$ $10^{13}$ vg/kg, and $4.5 \times 10^{13}$ vg/kg. Animals were euthanized one month after AAV vector administration. Skeletal muscles were dissected out and frozen in isopentane cooled in liquid nitrogen. Transverse cryosections (8 mm thickness) were prepared from frozen muscles, air dried, and stored at -80$^{\circ }$C. HE staining was performed on all sampled muscles. Whole section images of HE staining were acquired on Axioscan Z1 (ZEISS, Germany), using a plan-apochromat 10 $\times$ magnitude 0.45 NA objective (ZEISS, Germany). Pyramidal images were reconstructed using ZEN software (ZEISS, Germany). The study was performed in compliance with the ARRIVE guidelines.

### Dataset and ground truth creation

The dataset used in the context of this article is constituted of WSI of 50 mice having undergone the protocol described above. It is composed of WSI of mouse right psoas sections with 10 sections coming from 10 different Wild Type subjects, 10 from Knockout subjects, 10 from subjects treated with the lowest dose, 10 from those treated with intermediate dose and last 10 treated by the highest dose.

The ground truth segmentation was performed manually on three WSI of psoas sections on an area of about 200–314 ${\upmu }$m$^{2}$ using QuPath annotation capabilities.

### Scripts description and usage

To perform automated analysis of MyoSOTHES, two python programs, several groovy QuPath-compatible and shell scripts have been implemented.

Python scripts are written in python 3.8. The first script dealing with the segmentation stage uses 2.0.4 Cellpose version with numpy version 1.21.6 and PyTorch 1.11.0. As execution parameters, the input cell diameter and the input channel vary according to configuration studied. In all models, the flow threshold is set to 0.5 and the resampling option is set to true. The second python script modifies tile masks obtained for reconstruction, as described further in “Tiling and reconstruction strategy” subsection. It requires Pillow library version 8.0.1.

Concerning groovy scripts, they have been produced using 0.3.1 version of QuPath. A first script aims at performing automatically the export of histological whole slide image into TIFF images to be compatible with Cellpose input format. In case of tiling, a script is available to export tiles containing tissue. Another script enables to superimpose Cellpose predicted masks on corresponding sections and assign them the prediction class. This script is available in two versions: the one simply importing one WSI mask, and a second version importing each tile mask and placing them at the right place before removing nested masks using “intersect” function from QuPath. Then, the following script to be executed is the one filtering imported masks. It executes a binary pixel classifier, based on a random forest, trained considering Gaussian, Laplacian of Gaussian and weighted deviation features, on red, green and blue channels, at scale 1.0. Once masks filtered, their Feret’s diameter is measured using the QuPath minimum diameter function accessible via the “Adding shape characteristics” option. Once Feret’s diameter estimated, CNF ratio can be computed. To do so, the cell detection plugin with cell expansion set to 0 is applied, which detects nuclei in the entire image and stores them as a detection object. Then, for each mask, the hierarchy between regions of interests and detection is resolved, to assign which nuclei belong to which cell. The cell and nuclei boundaries are converted into polygons to measure the distance between nuclei boundary and cell boundary. If the distance is higher than a threshold of 2.2 $\upmu$m, we consider that the cell is centrally nucleated. Those two operations are run using one script measuring biological parameters. To perform error estimation, a script is produced to enable the import of ground truth masks stored in a GeoJSON format, which is the one produced by QuPath to export annotations. A final script provides the tools to compute IoU for True Positive, False Positive and False Negative detection, which are then interpreted for the estimation of recall, precision and F1-score. All QuPath tools used in script can be adapted to specific cases following the well detailed QuPath user guide available online^22^.

### Computation support specification

Cellpose computation time measurements were performed using three different machines. The first one is a computer NUC10i7FNH equipped with an Intel Core i7-10710U 1.10GHz processor, with 6 cores and 32 GB of RAM (Intel, USA). It executes Linux 5.13.0 with gcc 9.4.0, glibc 2.31. The second machine is a a computer OptiPlex 7060 having an Intel Core i5-8400 2.80GHz processor with 6 cores and 32 GB of RAM (Dell Inc., USA). It runs Linux 5.4.0 with gcc 9.4.0, glibc 2.31. The last one is a server AS -2023US-TR4 embedding 2 AMD EPYC 75022.5GHz processors with 32 cores and 512 GB of RAM (Supermicro, USA) and a Nvidia Quadro RTX 5000 GPU with 16 GB of memory (NVidia, USA). Linux 5.8.0 with gcc 8.4.0, glibc 2.31 and Cuda 11.2 are installed.

### Segmentation quality measure

The measurement of the segmentation quality must be considered from two angles. Indeed, to have a good quantification of some parameters such as the diameter, it is necessary to be able to detect the largest quantity of fiber possible to avoid biasing the population of detected fiber and also to ensure that the detected fiber has good quality contours. The model detection ability is measured based on F1-score metrics and contour quality metric is based on the measured parameter Root Mean Squared Error (RMSE). Both metrics rely on a previous metric, the Intersection over Union (IoU). IoU estimates whether a predicted mask can be considered as True Positive, that is, if the predicted mask corresponds to the ground truth according to a chosen threshold. If no corresponding ground truth mask exists, the predicted mask is considered as False Positive. On the contrary, when no predicted mask corresponds to a ground truth mask, it is labeled as False Negative. Those definitions are illustrated in Supplementary Fig. S2. The IoU threshold is required to be at least strictly higher than 0.5 to ensure at most one predicted mask corresponds to ground truth. The computation of IoU proposed here is performed in three stages. First, both predicted and ground truth masks have to be imported, assigning them a different class to differentiate them. The second stage consists in iterating over masks classified as predicted. They are all initially assigned the label False Positive. For each of them, the bounding box boundaries are determined. Then, the algorithm looks for any ground truth mask bounding box intersecting our current predicted masks, using QuPath “intersect” function between regions of interest. For intersecting masks, the IoU is computed based on intersection and union between regions of interest. The highest IoU between all intersecting ground truth masks is kept. If this value is higher than the preliminary fixed threshold, the current predicted mask label is changed to True Positive. The third stage consists in iterating over ground truth masks, initially labeled as False Negative. Same operations are performed switching prediction masks and ground truth masks, except that when IoU value is higher than the threshold, the attribution of the label False Negative is removed and not replaced. In the context of this study, the threshold is set to 0.5, as often selected by famous segmentation challenges like the Coco Challenge^18^ or the Voc Challenge^19^. Then, based on the class allocated to each mask, it is possible to measure the two other evaluation metrics. The F1-score is computed based on two indicators, precision and recall. The precision aims at estimating how many predicted masks correspond to a ground truth mask among all predicted masks, meaning how correct the predictions are. The recall estimates how many ground truth masks have a corresponding predicted mask among all ground truth masks, meaning how exhaustive the predictions are in the space of ground truth masks. The F1-score aims at embedding both dimensions by performing the harmonic mean between the two previous operators. The RMSE is computed among true positives. On each predicted and ground truth masks is performed the target parameter measurement. Then, the squared difference between corresponding predicted and ground truth parameter measurement is performed, as part of the root mean squared error formula $RMSE = \sqrt{(\frac{1}{n})\sum _{i=1}^{n}(\hat{p}_{i} - p_{i})^{2}}$ with $\hat{p}$ the measurement of target parameter on predicted masks, p the measurement of target parameter on ground truth masks and n the number of true positives.

### Cellpose retraining strategy

The retraining strategy is the one described in^17^. In total, 9 tiles extracted from different whole slide images were used, each containing the highest diversity as possible of HE specific artifacts and characteristics, such as inflammatory areas. Each tile contained about a hundred cells segmented. The extracted tiles used for training belong to different whole slide images than the ones used for segmentation quality estimation.

### Tiling and reconstruction strategy

The tiling strategy consists in dividing the WSI in patches and analyzing only patches containing tissues. The selection of tile containing tissue is done in QuPath using a thresholder, which turns out to be a subcategory of a pixel classifier. The classifier was trained with the following parameters: the resolution was set to low, meaning 7 $\upmu$m/pixel, the Gaussian prefilter was selected, the channel considered was the maximum of all channels and the threshold was set to 190, with 0 as smoothing sigma parameter. The region delimited by the thresholder is converted to an annotation. Then, the annotation itself is split into tiles, using the tiling tools offered by QuPath, embedding tile position in the extracted tile name. However, tiling induces side effects: fibers cut by tile limit are segmented less precisely and corresponding masks contain a straight edge. To alleviate this problem, tiling is performed extracting overlapped tiles. The overlapping size is set to 200 pixels to ensure no fibers are cut on image in the overlapping area they belong to. The detailed process is represented in Supplementary Fig. S3.

As a consequence, the reconstruction strategy is not trivial. The resulting Cellpose masks are retrieved in a PNG image format where each pixel, belonging to the same masks, has the same specific value. Consequently, to reconstruct the WSI masks without overlapping masks, we propose a python script which modifies tile masks as presented in Supplementary Fig. S4:

1. All masks belonging to upper and left 200 pixels width stripe will be removed by setting all pixel values to 0, except for those intersecting with lines x = 200 pixels and y = 200 pixels.
2. All masks cut by right and lower tile border are removed by replacing their pixel values by 0.

The final step is to place each mask at the corresponding position on the WSI using the position stored in image name, which is made available in a QuPath script. Then, in some marginal cases, two masks are nested due to side effects. To avoid overrepresentation of one mask, the largest of the two nested masks is kept. The detection is performed thanks to the QuPath “intersect” function between annotations.

## Supplementary Information

Supplementary Information.

## Data Availability

Code available on GitHub: https://github.com/brunettsp/myosothes. The dataset used and analysed during the current study is available from the corresponding author on reasonable request.
