# Supplementary material for: Artificial intelligence workflow quantifying muscle features on Hematoxylin–Eosin stained sections reveals dystrophic phenotype amelioration upon treatment
Source: Sci Rep. 2022 Nov 19;12:19913. doi: 10.1038/s41598-022-24139-z (PMC9675753; doi:10.1038/s41598-022-24139-z)
Supplement: Supplementary file 1 — Supplementary Information. [file 41598_2022_24139_MOESM1_ESM.pdf]

Supplementary information

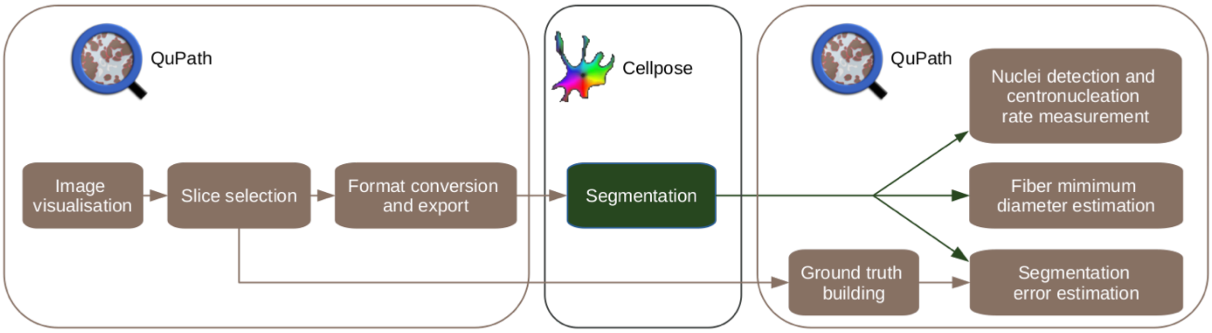

Supplementary Figure 1. Initial analysis workflow, composed of three main blocs: creation of dataset to be analyzed using QuPath, fiber segmentation performed with Cellpose, and phenotype quantification together with error estimation based on ground truth manual segmentation.

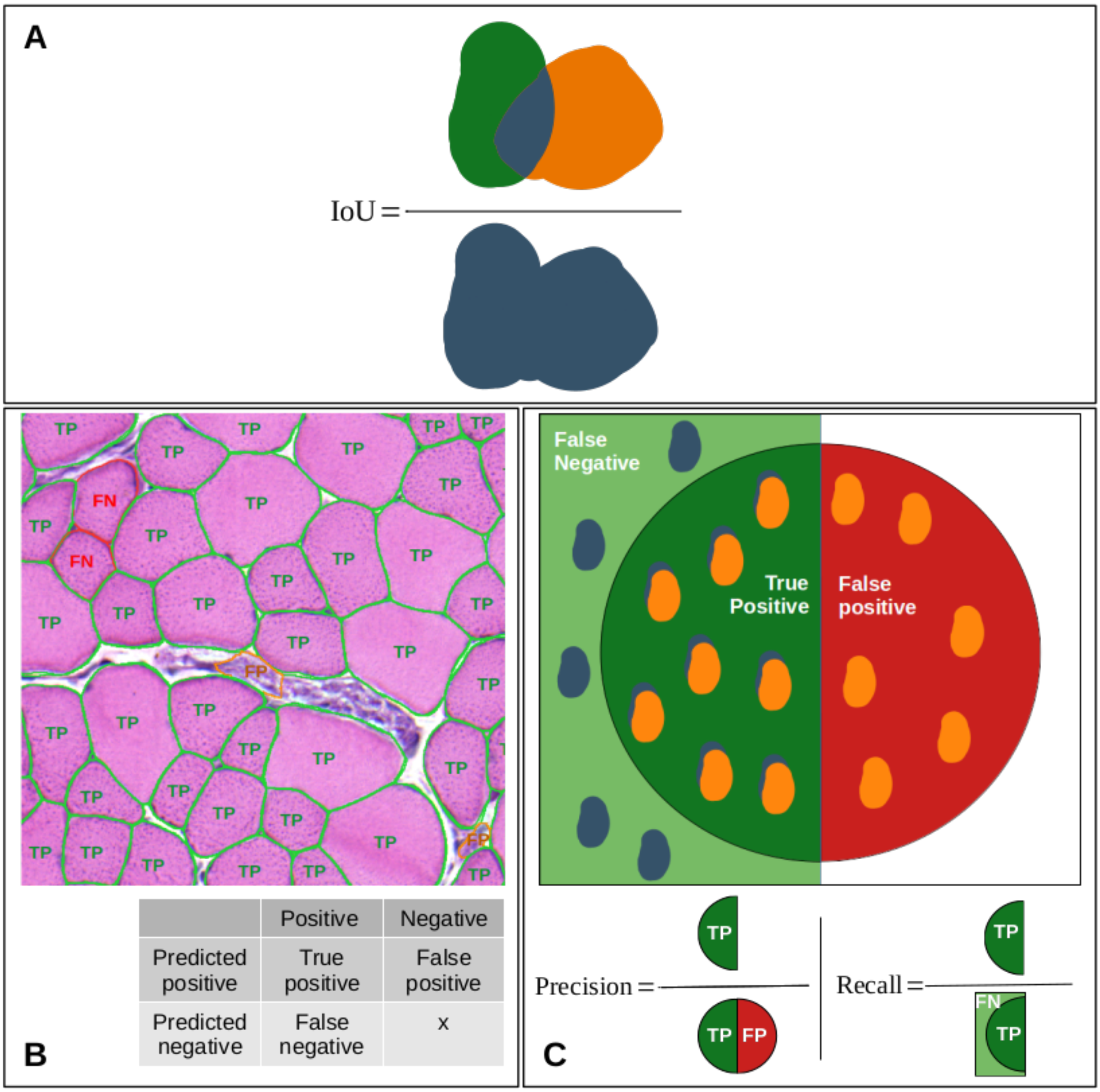

Supplementary Figure 2. Metrics for segmentation error estimation. A. Intersection over Union (IoU) graphic representation. B. Visual representation of True Positives (TP), False Positives (FP) and False Negatives (FN) in the context of muscle cells segmentation. C. Graphic representation of precision and recall for F1-score measurement, used as indicators to assess models performances.

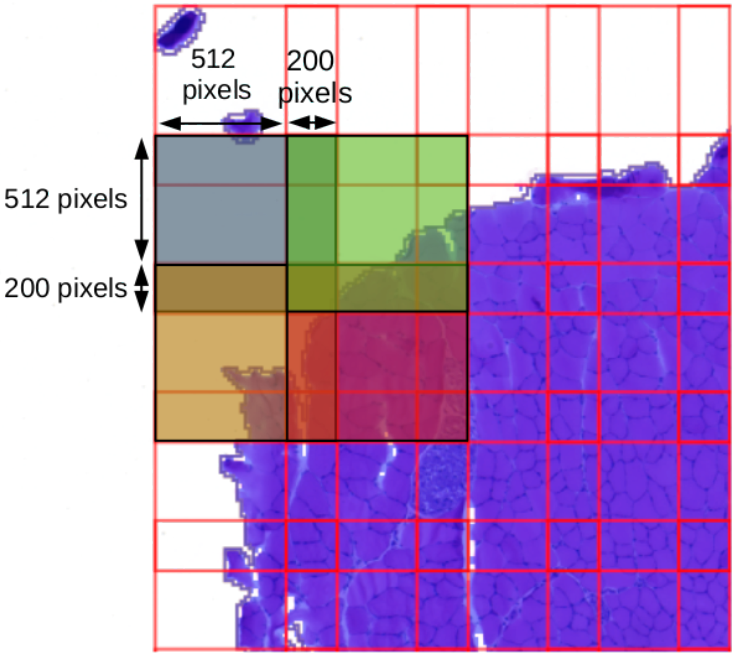

Supplementary Figure 3. Muscle section tiling strategy. Image tiled into patches of 712x712 pixels including 200 pixels overlapping with neighbour patches.

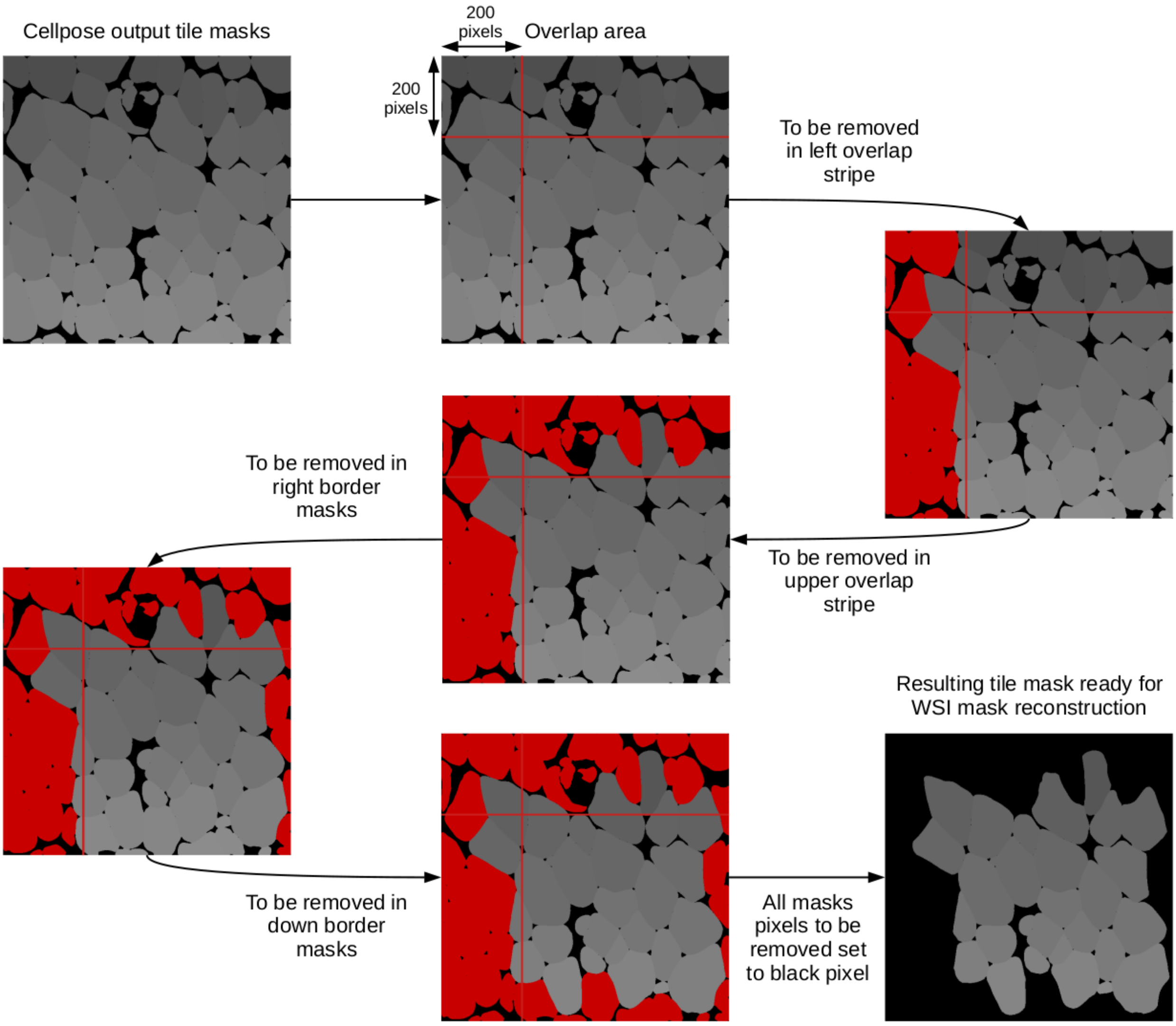

Supplementary Figure 4. Tiled masks modification prior to WSI mask reconstruction. Masks belonging to the two 200 pixels overlapping areas (left and upper stripes) are removed, except for masks intersecting with 200 pixels vertical and horizontal lines. Masks intersecting with edge of image (bottom and right) are removed. Modified patches are reassembled according to their location in original image to form WSI mask.

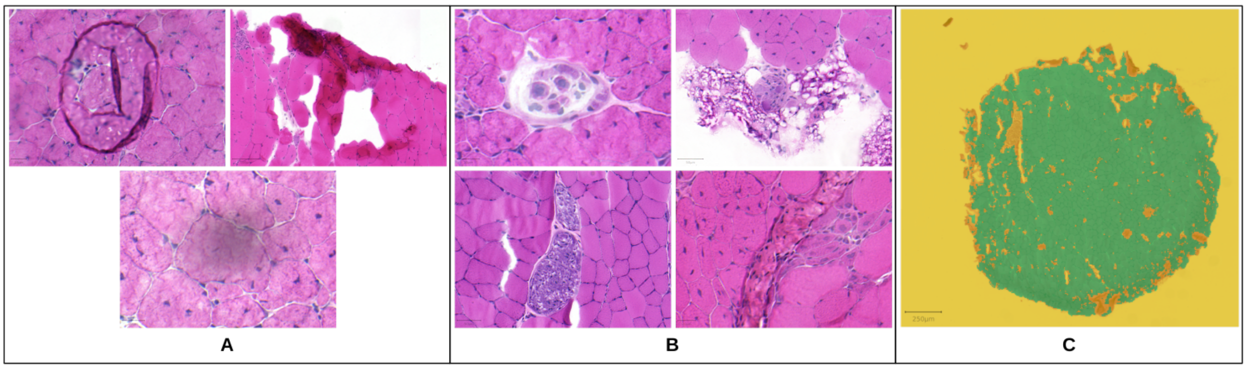

Supplementary Figure 5. Examples of artifacts often encountered on histopathological images. A. Representative images of histological artifacts (bubbles, tissue folds, dust, etc.). B. Representative images of tissue artifacts (blood vessel, lymphatic vessel, connective tissue, fibrosis, etc.). C. Results of pixel classifier-based artifact exclusion process. The muscle fiber analysis is run on areas displayed in green.
